# Precision Care in Screening, Surveillance, and Overall Management of Barrett’s Esophagus

**DOI:** 10.3390/jpm15080327

**Published:** 2025-07-22

**Authors:** Yeshaswini Reddy, Madhav Desai, Bernadette Tumaliuan, Nirav Thosani

**Affiliations:** 1 Interventional Gastroenterology at UT, The University of Texas Health Science Center at Houston, Houston, TX 77030, USA; bernadette.tumaliuan@uth.tmc.edu (B.T.); nirav.thosani@uth.tmc.edu (N.T.); 2 Borland Groover Clinic, Jacksonville, FL 32216, USA; mdesai@borlandgroover.com

**Keywords:** precision medicine, Barrett’s esophagus, radiofrequency ablation, cryotherapy, intestinal metaplasia, low-grade dysplasia, high-grade dysplasia

## Abstract

Barrett’s esophagus (BE), a metaplastic transformation of an esophageal squamous epithelium into an intestinal-type columnar epithelium, is the primary precursor to esophageal adenocarcinoma (EAC). Traditional management strategies have relied heavily on selective screening, tailored surveillance intervals, and early dysplasia detection and treatment algorithms. However, the heterogeneity in progression risk among BE patients necessitates a more nuanced, personalized approach involving precision care, tailoring decisions to individual patient characteristics, promises to enhance outcomes in BE through more targeted screening, personalized surveillance intervals, and risk-based therapeutic strategies. This review explores the current landscape and emerging trends in precision medicine for Barrett’s esophagus, highlighting genomic markers, digital pathology, and AI-driven models as tools to transform how we approach this complex disease and prevent progression to EAC.

## 1. Introduction

Barrett’s esophagus (BE), a precursor of esophageal adenocarcinoma (EAC), has been diagnosed in approximately 1–2% of the general population and 5–15% of patients with a history of gastroesophageal reflux disease. In BE, the normal squamous distal esophageal mucosa is replaced by metaplastic intestinal-type columnar cells [1,2]. BE progresses to EAC in a stepwise fashion from metaplasia to low-grade and then high-grade dysplasia/intra-mucosal carcinoma to, finally, invasive EAC. Therefore, the early diagnosis and management of BE decrease the risk of progression to EAC, which has an increasing incidence and a dismal 5-year survival rate of <20% [3]. While the annual progression rate from non-dysplastic BE (NDBE) to EAC remains relatively low (0.1–0.3%) [4], the benefit of the intensity of surveillance and treatment to the population remains debatable. Progression to EAC also depends on patient factors, including the control of acid reflux, smoking status, and the degree of dysplasia, as well as the endoscopist’s accuracy, technical expertise, risk assessment, and treatment for each patient. While there have been advances in the endoscopic detection of BE and related dysplasia, novel non-invasive tools to help diagnose dysplasia early are essential. Traditional management relies on population-based guidelines for screening and surveillance, which can lead to the over- and underutilization of resources. With advances in molecular diagnostics, imaging, and computational analytics, precision care offers a new paradigm to tailor management based on individualized risk. This concept helps customize available tests and select the best therapy, including guidelines using the patient’s genetic, molecular, or cellular assessments. Precision medicine (PM) in BE could help to practice cost-efficient and effective treatments that are individualized for each patient, ultimately resulting in EAC elimination [1]. A lack of prospective validation for many emerging biomarkers and infrastructure needs for AI integration limits the implementation of precision care in BE. Ongoing efforts are needed to create interoperable databases, longitudinal cohorts, and multicenter registries. Regulatory and ethical considerations will be paramount as personalized approaches become standard.

## 2. Precision Screening Strategies: Who and When?

### 2.1. Risk-Based Screening

Current screening criteria for BE include chronic gastroesophageal reflux disease (GERD) symptoms, age > 50, male sex, central obesity, Caucasian race, family history of EAC/BE, and smoking history. All major U.S. societies (AGA, ACG, ASGE) endorse risk-stratified screening for BE rather than universal screening [2,5,6]. The ACG 2022 guideline on this topic recommends screening for men over 50 with chronic GERD and at least three additional risk factors (obesity, Caucasian race, smoking, family history of BE/EAC) [5]. The ASGE 2019 guideline on this topic adopts a broader threshold, recommending screening for at-risk populations, defined as individuals with a family history of EAC or BE (high risk) or patients with GERD plus at least one other risk factor (moderate risk) [2]. However, these broad criteria can fail to identify up to 40% of patients with BE, especially those with silent reflux or non-traditional risk factors [1].

### 2.2. Emerging Non-Endoscopic Tools and Biomarkers

Emerging precision approaches incorporate biomarkers, such as serum trefoil factor 3 (TFF3), and cytological assessment using minimally invasive tools, like the Cytosponge. The Cytosponge-TFF3 test gained attention following the BEST3 trial, demonstrating a tenfold increase in BE detection compared to standard care [7]. Genetic predisposition (e.g., *FOXF1*, *CRTC1* polymorphisms) and polygenic risk scores (PRSs) are being explored to refine screening eligibility. Integrating demographic, clinical, and molecular data via machine learning models may enhance risk prediction. ACG and AGA guidelines acknowledge these tools as viable alternatives for selected populations [2,5].

In the EMERALD international multicenter study, researchers developed and validated a novel six-microRNA blood-based assay for the early detection of esophageal adenocarcinoma (EAC) and Barrett’s esophagus (BE). Using 792 patient samples, they identified a consistent six-miRNA signature that accurately distinguished affected individuals from controls, achieving AUROCs of 97.6% in training and 91.9% in validation cohorts. The assay also detected BE among GERD patients (AUROC: 94.8%). This liquid biopsy shows promise as a noninvasive screening tool to complement current strategies for BE and EAC detection [8]

Another non-endoscopic innovation developed recently is the EsoCheck/EsoGuard platform, which utilizes a swallowable balloon capsule to collect esophageal cells for methylated DNA biomarker analysis (VIM and CCNA1). This is the only FDA-approved commercial tool for esophageal sampling, has demonstrated high diagnostic accuracy in multiple trials, and was suggested as an alternative in the ACG guidelines [9,10]. These large multicenter studies assessed its role in high-risk asymptomatic individuals. For surveillance and risk stratification, the Barrett’s Aneuploidy Decision (BAD) classifier integrates esophageal brushing-derived aneuploidy scores with key chromosomal alterations (e.g., 9p loss, 8q24 gain) to categorize BE lesions into progression risk tiers. In validation studies, the BAD classifier identified 96% of esophageal adenocarcinomas and showed strong predictive performance for dysplasia progression over a 36-month follow-up [11]. These tools, alongside emerging technologies like TissueCypher, represent a significant shift in BE management, enabling earlier, more accurate detection and individualized surveillance beyond the limitations of random biopsy protocols.

### 2.3. Diagnostic Criteria and Surveillance Intervals

#### 2.3.1. Histologic Confirmation and Dysplasia Grading

All societies require endoscopic evidence of columnar-lined epithelium and histologic confirmation of intestinal metaplasia for a BE diagnosis. Due to high interobserver variability, dysplasia is graded as non-dysplastic BE (NDBE), indefinite for dysplasia, low-grade dysplasia (LGD), or high-grade dysplasia (HGD), with confirmation by expert GI pathologists [5,12].

#### 2.3.2. Surveillance Intervals

- NDBE: Every 3–5 years; ACG stratifies by segment length (3 years for long segment, 5 years for short segment).
- Indefinite for Dysplasia: Repeat endoscopy in 3–6 months post-PPI therapy.
- LGD: Preferably treated with endoscopic eradication therapy (EET); if not, 6–12 months surveillance.
- HGD: Immediate EET; surveillance is not recommended unless the patient is unfit for therapy.

### 2.4. Limitations of Current Surveillance

Standard guidelines recommend surveillance endoscopy every 3–5 years for non-dysplastic BE (NDBE) and more frequent intervals for dysplasia. This approach assumes homogeneous risk progression across patients, which is not supported by longitudinal studies.

### 2.5. Molecular and Genomic Risk Stratification

Genomic alterations such as TP53 mutations, aneuploidy, and DNA methylation patterns are strong predictors of progression to EAC [3]. Incorporating these into surveillance algorithms may allow clinicians to lengthen intervals for truly low-risk patients and intensify monitoring for high-risk individuals.

### 2.6. Artificial Intelligence and Digital Pathology

AI-based image analysis systems are being trained to detect early dysplasia with accuracy comparable to expert pathologists. AI has also demonstrated utility in standardizing biopsy grading and reducing interobserver variability, particularly in low-grade dysplasia [13].

### 2.7. Individualized Therapeutic Management

Endoscopic Eradication therapy where goal is to remove any visible lesion in the segment of Barrett’s esophagus to diagnose, stage and potentially cure any early cancer followed by elimination of flat dysplasia and remaining Barrett’s epithelium by ablation is the mainstay of treatment.Endoscopic resection (EMR/ESD) and radiofrequency ablation (RFA)-based endoscopic eradication therapy is the standard of care for patients with dysplastic BE. Precision care supports tailoring interventions based on molecular profiles that predict response and recurrence risk.

## 3. Management of Barrett’s Esophagus Related Dyspla-Sia/Neoplasia

### 3.1. Endoscopic Therapy

#### 3.1.1. Role of Endoscopic Ablation

Radiofrequency ablation (RFA) remains the standard of care for ablating flat dysplasia BE mucosa. The AIM-II trial showed a >90% eradication of dysplasia and significantly reduced cancer progression [14]. Balloon-based cryotherapy has emerged as an alternative for refractory cases or patients with anatomical challenges. Studies report similar eradication rates (~80–90%) and lower stricture rates [15,16]. Guidelines do not state a preference but endorse both as acceptable therapies. Spray cryotherapy, which uses liquified nitrogen or carbon dioxide to freeze and destroy the abnormal epithelium, has gained traction, particularly in cases refractory to RFA or when deeper tissue penetration is needed. Spray cryotherapy also preserves the extracellular matrix, possibly contributing to lower stricture rates. While long-term outcomes are still under evaluation, early data suggest comparable efficacy with possibly fewer sessions [17,18]. The choice between modalities may reflect anatomical, histologic, and even logistical considerations [6]. Currently used endoscopic ablation modalities are shown in Table 1.

#### 3.1.2. Role of Endoscopic Resection

**Figure 1:**
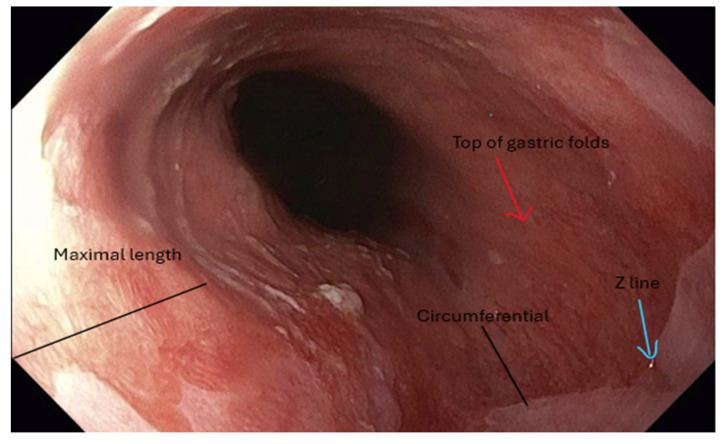
Endoscopic image demonstrating salmon-colored mucosa characteristic of Barrett’s esophagus. The distance from the top of the gastric folds to the Z-line represents the circumferential extent of BE. At the same time, the maximal length is defined by the most proximal extension of the salmon-colored mucosa.

**Figure 2:**
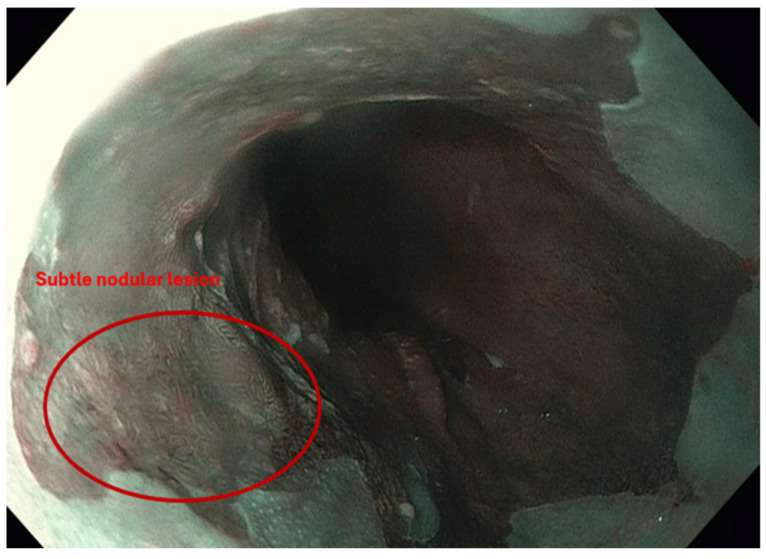
Narrow-band imaging (NBI) highlights a subtle nodular lesion within a background of Barrett’s esophagus. The lesion demonstrates mucosal irregularity suggestive of dysplasia or early neoplasia.

Endoscopic resection is indicated for BE with nodular or visible lesions and serves diagnostic and therapeutic purposes [2]. High-definition endoscopic inspection is essential to identify subtle mucosal abnormalities that may harbor early neoplasia. In cases of mixed flat and nodular disease, submucosal invasion or poorly differentiated carcinoma may be present, necessitating esophagectomy. A study from the Netherlands reported that 76% of visible lesions were detected only on repeat endoscopy performed by expert endoscopists, highlighting the critical role of experience in lesion recognition [19]. Figure 1 shows images of BE and a subtle lesion, and Figure 2 shows BE and a lesion on NBI. Following resection, ablative therapy is recommended to eradicate residual Barrett’s epithelium and reduce recurrence risk. Comparative studies have shown that both cap-assisted and band ligation EMR techniques are safe, each with a 5% perforation rate, though band ligation is faster. Endoscopic submucosal dissection (ESD) enables en bloc and R0 resection for larger or complex lesions, albeit with increased technical demands and procedural time [6]. Currently used endoscopic resection modalities are shown in Table 2.

#### 3.1.3. Post-Eradication Surveillance

Patients who achieve complete eradication of intestinal metaplasia require lifelong surveillance due to the risk of recurrence (approximately 20–30% at 5 years). ACG recommends endoscopies at 3, 6, and 12 months post-ablation and every 6–12 months thereafter, depending on the initial dysplasia grade.

### 3.2. Lifestyle, Diet, and Risk Modulation

Central obesity, high-fat diets, and smoking are established risk factors. The Seattle BE Study found increased vegetable intake to be inversely associated with BE risk [20]. Dietary patterns rich in red meat and low in fruits and vegetables have been linked to an increased risk of BE. Antioxidant-rich diets, particularly those high in vitamins C, E, and β-carotene, may offer protective effects. The frequent consumption of carbonated beverages, fried foods, and acidic items can exacerbate reflux and inflammation, thereby compounding the risk [20]. Personalized dietary counseling, informed by symptom patterns and nutritional status, is a low-cost but often underutilized tool. Weight loss and acid suppression with PPIs are standard recommendations. While precision care often involves high-tech tools, a truly holistic approach also considers modifiable lifestyle factors, particularly diet and obesity, which significantly influence BE risk and progression. Central obesity is a well-established risk factor for Barrett’s esophagus (BE) and esophageal adenocarcinoma (EAC), independent of GERD symptoms. Adipose tissue promotes a pro-inflammatory microenvironment and increases intra-abdominal pressure, which can exacerbate reflux. A 2015 meta-analysis by Singh et al. demonstrated a strong correlation between visceral fat and BE prevalence, even in patients with normal BMI [21].

### 3.3. Dysplasia Detection

#### 3.3.1. Role of Image Enhanced Endoscopy

High-definition white-light endoscopy combined with virtual chromoendoscopy (e.g., NBI) improves dysplasia detection and is recommended by all relevant societies [6]. The Barrett’s International NBI Group (BING) developed and validated a consensus-driven narrow-band imaging (NBI) classification system to identify dysplasia and EAC in BE. Using mucosal and vascular patterns observed in 60 initial NBI images, followed by validation on 170 additional images across U.S. and European centers, the BING criteria demonstrated 85% overall accuracy, with a sensitivity and specificity of 80% and 88%, respectively. When dysplasia was identified with high confidence, accuracy exceeded 90% and inter-observer agreement was substantial. This system offers a simple and reliable tool for dysplasia detection in BE [22].

Recent studies have demonstrated the utility of HSI combined with AI-based algorithms in enhancing diagnostic precision. For instance, Lin et al. in 2022 introduced the Spectrum-Aided Visual Enhancer (SAVE) algorithm, which simulates narrow-band imaging (NBI) from standard white-light images, resulting in improved precision and F1-scores without the need for dedicated NBI equipment. Wang et al. in 2024 validated a YOLO-based neural network trained on 1836 images, showing diagnostic accuracies of 0.90 (WLI) and 0.89 (NBI) with HSI—significantly surpassing RGB-based models. These findings underscore the potential of HSI and MSI technologies, particularly when paired with AI, to enhance the non-invasive, real-time detection of dysplasia and early-stage esophageal cancer [23,24].

#### 3.3.2. Role of WATS-3D and Tissue Sampling

WATS-3D, a computer-assisted brush biopsy tool, improves dysplasia detection when used adjunctively [2].

#### 3.3.3. Role of Artificial Intelligence (AI)

AI-based systems for endoscopic image analysis and pathology interpretation show promise in the real-time detection of neoplasia [25]; however, they are not yet standard in clinical guidelines. The future integration of AI into endoscopic platforms may enhance precision.

##### AI in Endoscopic Detection

Several deep learning systems have been trained to detect neoplasia in real time during high-definition endoscopy, with accuracy rivaling or exceeding that of human experts [25]. These tools can highlight suspicious areas, aiding targeted biopsies and reducing miss rates.

##### AI in Pathology

Digital pathology platforms are using convolutional neural networks to grade dysplasia, minimize interobserver variability, and even quantify subtle changes invisible to the naked eye. Various large data studies so far provide robust evidence supporting the accuracy to help diagnose and classify the lesions in a timely manner [13].

### 3.4. Emerging Research

There is growing interest in microbiome composition and its role in esophageal inflammation and the development of neoplasia. Future precision care may include modulating the microbiome through diet or probiotics.

### 3.5. Predictive Models

Machine learning algorithms that integrate clinical, endoscopic, and genomic data are being developed to stratify the risk of progression beyond current guidelines. These tools could one day guide the frequency of surveillance or the selection of therapeutic modalities. At the 2024 American Foregut Society meeting, two studies validated the TissueCypher TSP-9 test as a robust stand-alone predictor of neoplastic progression in Barrett’s esophagus. In a pooled analysis of 683 patients, the TSP-9 score outperformed models that included additional clinical or pathologic factors, which did not enhance and sometimes reduced predictive accuracy [26]. The systems biology-based test, which assesses multiple tissue compartments, categorizes patients as having a low, intermediate, or high risk of progression within five years, offering an objective tool to guide clinical decision-making.

### 3.6. Future Tools

- AI-assisted cytology from minimally invasive devices like the Cytosponge.
- Real-time risk calculators embedded in endoscopy software.
- Predictive analytics to personalize treatment response.

The challenge will be ensuring transparency, avoiding algorithmic bias, and maintaining clinician oversight.

## 4. Discussion

**Figure 3:**
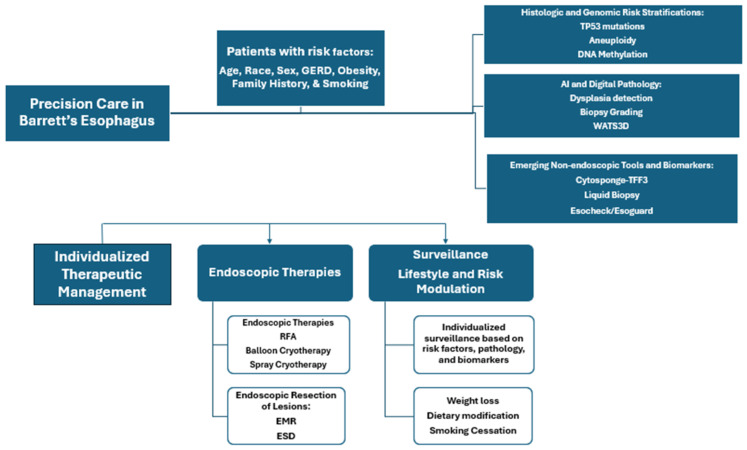
Precision medicine framework for improving care for patients with Barrett’s Esophagus.

Barrett’s esophagus (BE) is a premalignant transformation of the distal esophageal epithelium resulting from chronic gastroesophageal reflux disease (GERD). BE affects approximately 1.6% of adults and is characterized by the replacement of a normal squamous epithelium with specialized intestinal metaplasia (IM), the only known precursor to EAC. While the progression rate to esophageal adenocarcinoma (EAC) is relatively low, the high mortality associated with EAC necessitates early detection and individualized management [3]. However, the clinical course of BE is highly variable. While many patients remain stable, a subset progresses to dysplasia and EAC. Here, we outline the transformation of normal esophageal mucosa to EAC. Precision medicine could help improve current approach as shown in Figure 3.

### 4.1. Transition of Squamous Mucosa to Intestinal Metaplasia

BE arises at the gastroesophageal junction (GEJ), a transition zone between acid-sensitive squamous mucosa and oxyntic gastric mucosa. This buffer zone is typically less than 4 mm long. BE development occurs in two primary steps, namely chronic repetitive acid and bile reflux, often due to GERD, hiatal hernia (HH), incompetent LES, or erosive esophagitis, which induces injury and inflammation. Over time, the squamous mucosa is replaced by a columnar epithelium that resembles cardiac-type mucosa. This process can occur circumferentially or as tongues extending proximally [3]. The columnar epithelium may further differentiate into goblet cell-containing intestinal-type mucosa, a hallmark of Barrett’s esophagus (BE). This process is driven by environmental insults, such as bile acids and pH fluctuations, and genetic predisposition over a period of 5–10 years. Oxyntocardiac mucosa, characterized by parietal cells beneath cardiac glands, may be protective against IM. In contrast, intestinal gene expression (e.g., Cdx2) promotes the development of goblet cells and the intestinalization of the epithelium [3,19].

### 4.2. Role of Bile Salts and Inflammation

Acid reflux alone is insufficient to cause IM. Co-exposure to bile acids, particularly when the luminal pH ranges from 3 to 6, facilitates bile salt solubilization and epithelial injury [3]. These bile salts enter non-ionized, disrupt cellular homeostasis, and initiate carcinogenic pathways. Inflammation plays a critical role in BE pathogenesis. Elevated levels of pro-inflammatory cytokines, such as IL-1, have been found in BE patients. Chronic inflammation promotes increased cellular turnover, reduced apoptosis, and the accumulation of genomic instability [3,19].

### 4.3. Molecular Progression to Dysplasia and Cancer

Histologic progression in BE follows a dysplasia–carcinoma sequence:

- **No dysplasia → Indefinite → Low-grade dysplasia (LGD) → High-grade dysplasia (HGD) → EAC.**
- Dysplasia is defined by cytologic atypia, architectural distortion, and genetic aberrations.

Key molecular features include

- **Abnormal DNA content** (aneuploidy, tetraploidy).
- **Loss of tumor suppressors** (e.g., *p16*, *p53*).
- **Cdx2 gene expression** is often highest in IM.

The rate of malignant progression differs significantly as follows:

- LGD progresses to EAC at ~4% over 5 years.
- HGD progresses to EAC at ~61% over 5 years.

Due to substantial interobserver variability in dysplasia grading, particularly LGD, many institutions require expert GI pathology review. Molecular and genomic biomarkers are being increasingly evaluated to refine risk stratification [19].

### 4.4. Risk Factors and Epidemiology

Barrett’s esophagus exhibits a marked sex disparity, with a 2:1 male predominance. EAC is significantly more common in men, especially those over age 50 with chronic GERD and long-segment Barrett’s esophagus (LSBE) compared to women across age groups. Gender, however, does not affect the treatment outcomes of ablative therapies. Caucasians have the highest prevalence of BE, followed by Hispanics, with lower rates in Asians and African Americans. Obesity, particularly central adiposity, plays a significant role in the pathogenesis of GERD and BE and remains the most important modifiable risk factor. Increased BMI, waist-to-hip ratio (WHR), and abdominal diameter index (ADI) are independent risk factors for BE. WHR thresholds greater than 0.9 in men and greater than 0.85 in women are associated with a higher risk. Ineffective motility and a hypotonic lower esophageal sphincter (LES) increase esophageal acid and bile exposure, especially at night, accelerating mucosal injury and dysplasia. Regular NSAID use may reduce the risk of EAC, although its effect on BE development is unclear. A meta-analysis showed an inverse association between NSAID use and EAC, potentially due to reduced neoplastic progression in BE. Family history also contributes to BE risk, possibly via shared environmental and genetic factors, and may justify earlier screening in young adults with GERD. Dietary patterns, NSAID use, and chronic inflammation can all influence progression [1,2,3,4,5].

Hiatal hernia (HH) contributes to impaired function of the lower esophageal sphincter (LES), leading to acid reflux and mucosal injury. A meta-analysis of 33 studies confirmed HH as a significant risk factor for BE, especially LSBE. HH > 4 cm has been associated with higher EAC recurrence despite eradication therapy, possibly due to impaired esophageal clearance and more refractory reflux. Pear-shaped cryoballoon ablation systems may improve treatment outcomes in these patients. Erosive esophagitis (EE) is another established risk factor, increasing the incidence of Barrett’s esophagus (BE) fivefold. Treating EE can aid in dysplasia detection and facilitate safe endoscopic therapy. Notably, eosinophilic esophagitis (EoE) may coexist with BE, requiring biopsy proximal to the Barrett’s segment to avoid complications with ablation. Segment length correlates with cancer risk. One study reported dysplasia in 24% of LSBE patients compared to 8% in SSBE, with a 16% increased risk of EAC in LSBE. SSBE patients often exhibit upright reflux and better lower esophageal sphincter (LES) tone, whereas LSBE patients have more severe bipositional reflux [19,27]. Guidelines emphasize expert pathology confirmation of dysplasia due to high interobserver variability. A multicenter study showed that 73% of LGD cases were downstaged upon expert review [27].

### 4.5. Management of Dysplasia in Barrett’s Esophagus

Endoscopic eradication therapy (EET) is the recommended treatment for patients with confirmed low-grade dysplasia (LGD), contingent upon expert pathological review due to the high interobserver variability in diagnosis. Robust evidence supports this approach, with a pivotal study demonstrating that radiofrequency ablation (RFA) reduced progression to high-grade dysplasia (HGD) by 25% and to esophageal adenocarcinoma (EAC) by 7.4% over follow-up. In cases of HGD or intramucosal cancer (IMC), the standard of care includes the endoscopic resection of any visible or nodular lesions to facilitate accurate histologic staging and complete excision, followed by ablation of the residual flat Barrett’s epithelium to minimize recurrence risk. Importantly, lesion detection is operator-dependent; therefore, high-definition imaging and experienced interpretation are required to optimize outcomes [5,6,19].

### 4.6. Wide Accessibility of Precision Tools

To improve reach, we recommend prioritizing cost-effective, non-endoscopic sampling techniques such as the Cytosponge and EsoCheck, which have demonstrated feasibility and accuracy in primary care settings and can facilitate molecular testing without the need for high-cost endoscopy platforms [7,9]. We also highlight the value of developing lightweight AI algorithms that are deployable on mobile or cloud-based platforms, which can support clinical decision-making in areas lacking advanced computational infrastructure [28]. Open access datasets, global collaborations, and workforce training initiatives will also be essential. Health policy support and scalable deployment frameworks will be critical for the equitable global implementation of precision diagnostics.

We emphasize the importance of structured and specific training programs for gastroenterologists, pathologists, and endoscopy staff to facilitate clinical adoption. These programs should focus on the interpretation of molecular and methylation-based assays, the use of AI-enabled decision support tools during endoscopy, and the integration of such tools into guideline-based surveillance pathways. Continuing medical education (CME), simulation-based learning, and collaboration with professional societies (e.g., ACG, ASGE) are key to ensuring proficiency and sustained implementation. Prior studies have shown that such approaches improve diagnostic performance and clinician confidence in the context of BE-related technologies [26].

### 4.7. Validation of AI Models in Clinical and Real-World Settings

Despite significant progress in the development of AI-based tools for Barrett’s esophagus (BE), their widespread clinical adoption remains limited by the lack of prospective validation and real-world performance data. Several studies have demonstrated the potential of deep learning algorithms to accurately detect dysplasia and early esophageal adenocarcinoma from endoscopic images in retrospective settings. Ebigbo et al., 2019, reported the successful application of AI in differentiating early esophageal adenocarcinoma from non-neoplastic BE. However, these models require further validation through multi-center prospective clinical trials to assess their generalizability and integration into diverse healthcare environments. Establishing standardized evaluation protocols and incorporating AI tools into real-world endoscopy workflows will be essential to translating these advances into routine clinical care [29].

### 4.8. Ethical and Privacy Concerns in Genomic and AI-Based Tools

The ethical use of genomic and AI-based technologies in Barrett’s esophagus requires robust governance frameworks. Strategies include transparent informed consent, data de-identification, adherence to regulatory standards (e.g., HIPAA, GDPR), and the use of privacy-preserving techniques such as federated learning. These safeguards are crucial for protecting patient confidentiality while facilitating scalable and ethically responsible implementation [28,30].

Barrett’s is not a monolith but a spectrum disorder with widely varying trajectories. This shift from categorical staging to a risk continum is more than academic. It affects how we screen, how frequently we surveil, and whom we treat. One of the most striking insights from the past decade is the relatively low annual progression rate from non-dysplastic Barrett’s esophagus (BE) to cancer, estimated at 0.1–0.5% [4]. This raises ethical and financial concerns about blanket surveillance. Conversely, the minority who do progress often exhibit subtle biological signs of improvement well in advance. Precision care offers a solution to both sides of this paradox, decreasing unnecessary interventions in low-risk patients while accelerating care for those at the highest risk by tailoring screening, surveillance, and treatment to individual risk profiles. Integrating advanced diagnostics to improve early detection and outcomes is crucial. Differences among the AGA, ACG, and ASGE guidelines reflect evolving evidence, but they share a common emphasis on high-quality, patient-centered care. But the path to implementation is complex. The standardization of molecular tests, insurance coverage, and equitable access must all be addressed. To support the integration of genetic, non-endoscopic tools and molecular biomarkers into international guidelines, further large-scale, prospective, and standardized studies are needed to establish their clinical utility and enable a more personalized approach to the management of Barrett’s esophagus. Furthermore, patient preferences vary widely; some may prioritize aggressive monitoring for peace of mind, while others may prefer to avoid invasive procedures. These human elements must remain at the forefront of our approach. To ensure the generalizability of precision medicine tools in Barrett’s esophagus (BE), strategies must prioritize diversity, standardization, and global collaboration. This includes enrolling racially, ethnically, and socioeconomically diverse patient cohorts; harmonizing biomarker assays and AI algorithms; and validating tools across varied clinical settings using external datasets and real-world implementation studies. Cross-platform compatibility, data interoperability, and privacy-preserving federated learning are essential for scalable integration.

While precision medicine offers significant promise in enhancing early detection and individualized care in Barrett’s esophagus (BE), it also raises concerns regarding the potential for overdiagnosis, particularly with the integration of genomic and digital diagnostic technologies. The primary aim of precision strategies in BE is not to expand surveillance indiscriminately but to refine risk stratification—minimizing unnecessary interventions in low-risk individuals while focusing resources on those at the greatest risk of progression to high-grade dysplasia or esophageal adenocarcinoma. Emerging studies support this approach: Molecular tools such as methylation-based classifiers and genomic risk models have demonstrated the ability to improve diagnostic specificity and guide surveillance de-escalation in appropriate patients [7,9,31]. These findings highlight the importance of ongoing prospective validation studies, cost-effectiveness analyses, and patient-centered outcome research to ensure that the clinical implementation of precision tools delivers a net benefit while mitigating the risks of overtreatment and patient harm.

## 5. Conclusions

Precision medicine holds transformative potential in the management of Barrett’s esophagus. By moving beyond generalized guidelines to individualized care, clinicians can improve early detection, optimize surveillance strategies, and enhance therapeutic outcomes. Future research must focus on integrating molecular, digital, and clinical data into accessible, evidence-based decision tools.

## Figures and Tables

**Table 1:** Currently used endoscopic ablation modalities for treatment of Barrett’s Esophagus related dysplasia.

Feature	RFA	Spray Cryotherapy	Balloon Cryotherapy
**Mechanism**	Thermal energy	Rapid freezing using liquid nitrogen or CO_2_	Contact-based freezing via balloon catheter with nitrous oxide
**Depth of Tissue Injury**	Controlled, superficial	Deeper, potentially transmural	Intermediate—more uniform than spray, less than RFA
**Dysplasia Eradication Rate**	~90% for LGD/HGD	~80–90%	~88–92% for dysplasia eradication
**Complication Profile**	Strictures (5–10%), post-procedure pain	Minimal stricture, transient chest discomfort	Low stricture rate, mild transient discomfort
**Role in Practice**	First-line for most dysplasia	Salvage/rescue therapy or in high-risk anatomies or strictures	Emerging alternative; used in routine and rescue settings
**Technical Notes**	Requires close mucosal contact	Non-contact, variable application; good for uneven mucosa	More uniform freeze, easier dosing, better circumferential control

**Table 2:** Currently used endoscopic resection modalities for treatment of Barrett’s Esophagus related dysplasia.

Technique	Safety Profile	Procedure Time	Resection Quality	Technical Demand
**Cap-Assisted EMR**	~5% perforation risk	Slower	Piecemeal resection	Moderate
**Band Ligation EMR**	~5% perforation risk	Faster	Piecemeal resection	Moderate
**ESD**	Safe in experienced hands	Longer	En bloc, R0 resection	High (requires training)

## References

[1] Triadafilopoulos G., Friedland S. (2018). Precision care for Barrett’s esophagus. Transl. Gastroenterol. Hepatol..

[2] Evans J.A., Early D.S., Fukami N., Ben-Menachem T., Chandrasekhara V., Chathadi K.V., Decker G.A., Fanelli R.D., Fisher D.A., Foley K.Q. (2012). The role of endoscopy in Barrett’s esophagus and other premalignant conditions of the esophagus. Gastrointest. Endosc..

[3] Shaheen N.J., Richter J.E. (2009). Barrett’s oesophagus. Lancet.

[4] Desai T.K., Krishnan K., Samala N. (2012). The incidence of oesophageal adenocarcinoma in nondysplastic Barrett’s oesophagus: A meta-analysis. Gut.

[5] Han A.J. (2022). Continuing Medical Education Questions: March 2022. Am. J. Gastroenterol..

[6] Thosani N., Abu Dayyeh B.K., Sharma P., Aslanian H.R., Enestvedt B.K., Komanduri S., Manfredi M., Navaneethan U., Maple J.T., Pannala R. (2016). ASGE Technology Committee systematic review and meta-analysis assessing the ASGE Preservation and Incorporation of Valuable Endoscopic Innovations thresholds for adopting real-time imaging–assisted endoscopic targeted biopsy during endoscopic surveillance of Barrett’s esophagus. Gastrointest. Endosc..

[7] Fitzgerald R.C., di Pietro M., O’DOnovan M., Maroni R., Muldrew B., Debiram-Beecham I., Gehrung M., Offman J., Tripathi M., Smith S.G. (2020). Cytosponge-trefoil factor 3 versus usual care to identify Barrett’s oesophagus in a primary care setting: A multicentre, pragmatic, randomised controlled trial. Lancet.

[8] Miyoshi J., Mannucci A., Scarpa M., Gao F., Toden S., Whitsett T., Inge L.J., Bremner R.M., Takayama T., Cheng Y. (2024). Liquid biopsy to identify Barrett’s oesophagus, dysplasia and oesophageal adenocarcinoma: The *EMERALD* multicentre study. Gut.

[9] Greer K.B., Blum A.E., Faulx A.L., Deming E.M., Hricik L.L., Siddiqui H., Wilson B.M., Chak A. (2024). Nonendoscopic Screening for Barrett’s Esophagus and Esophageal Adenocarcinoma in At-Risk Veterans. Am. J. Gastroenterol..

[10] Shaheen N.J., Othman M.O., Taunk J., Chang K.J., Jaganmohan S., Yachimski P.S., Fang J.C., Spataro J.S., Verma S., Lee V.T. (2024). Use of the EsoGuard^®^ molecular biomarker test in non-endoscopic detection of Barrett’s esophagus among high-risk individuals in a screening population. medRxiv.

[11] Moinova H.R., Verma S., Dumot J., Faulx A., Iyer P.G., Canto M.I., Wang J.S., Shaheen N.J., Thota P.N., Aklog L. (2024). Multicenter, Prospective Trial of Nonendoscopic Biomarker-Driven Detection of Barrett’s Esophagus and Esophageal Adenocarcinoma. Am. J. Gastroenterol..

[12] Duits L.C., van der Wel M.J., Cotton C.C., Phoa K.N., Kate F.J.T., Seldenrijk C.A., Offerhaus G.J.A., Visser M., Meijer S.L., Mallant-Hent R.C. (2017). Patients With Barrett’s Esophagus and Confirmed Persistent Low-Grade Dysplasia Are at Increased Risk for Progression to Neoplasia. Gastroenterology.

[13] Cui R., Wang L., Lin L., Li J., Lu R., Liu S., Liu B., Gu Y., Zhang H., Shang Q. (2023). Deep Learning in Barrett’s Esophagus Diagnosis: Current Status and Future Directions. Bioengineering.

[14] Shaheen N.J., Sharma P., Overholt B.F., Wolfsen H.C., Sampliner R.E., Wang K.K., Galanko J.A., Bronner M.P., Goldblum J.R., Bennett A.E. (2009). Radiofrequency Ablation in Barrett’s Esophagus with Dysplasia. N. Engl. J. Med..

[15] Kochman M.L., McClave S.A., Boyce H.W. (2005). The refractory and the recurrent esophageal stricture: A definition. Gastrointest. Endosc..

[16] Canto M.I., Trindade A.J., Abrams J., Rosenblum M., Dumot J., Chak A., Iyer P., Diehl D., Khara H.S., Corbett F.S. (2020). Multifocal Cryoballoon Ablation for Eradication of Barrett’s Esophagus-Related Neoplasia: A Prospective Multicenter Clinical Trial. Am. J. Gastroenterol..

[17] Gondrie J., Pouw R., Sondermeijer C., Peters F., Curvers W., Rosmolen W., Kate F.T., Fockens P., Bergman J. (2008). Effective treatment of early Barrett’s neoplasia with stepwise circumferential and focal ablation using the HALO system. Endoscopy.

[18] Ghorbani S., Tsai F.C., Greenwald B.D., Jang S., Dumot J.A., McKinley M.J., Shaheen N.J., Habr F., Coyle W.J. (2016). Safety and efficacy of endoscopic spray cryotherapy for Barrett’s dysplasia: Results of the National Cryospray Registry. Dis. Esophagus.

[19] Orlando R.C. (2005). Pathogenesis of reflux esophagitis and Barrett’s esophagus. Med. Clin. N. Am..

[20] Conio M., Filiberti R., Blanchi S., Ferraris R., Marchi S., Ravelli P., Lapertosa G., Iaquinto G., Sablich R., Gusmaroli R. (2001). Risk factors for Barrett’s esophagus: A case-control study. Int. J. Cancer.

[21] Singh S., Sharma A.N., Murad M.H., Buttar N.S., El–Serag H.B., Katzka D.A., Iyer P.G. (2013). Central Adiposity Is Associated With Increased Risk of Esophageal Inflammation, Metaplasia, and Adenocarcinoma: A Systematic Review and Meta-analysis. Clin. Gastroenterol. Hepatol..

[22] Sharma P., Bergman J.J., Goda K., Kato M., Messmann H., Alsop B.R., Gupta N., Vennalaganti P., Hall M., Konda V. (2016). Development and Validation of a Classification System to Identify High-Grade Dysplasia and Esophageal Adenocarcinoma in Barrett’s Esophagus Using Narrow-Band Imaging. Gastroenterology.

[23] Lin T.-L., Karmakar R., Mukundan A., Chaudhari S., Hsiao Y.-P., Hsieh S.-C., Wang H.-C. (2025). Assessing the Efficacy of the Spectrum-Aided Vision Enhancer (SAVE) to Detect Acral Lentiginous Melanoma, Melanoma In Situ, Nodular Melanoma, and Superficial Spreading Melanoma: Part II. Diagnostics.

[24] Wang Y.-K., Karmakar R., Mukundan A., Men T.-C., Tsao Y.-M., Lu S.-C., Wu I.-C., Wang H.-C. (2024). Computer-aided endoscopic diagnostic system modified with hyperspectral imaging for the classification of esophageal neoplasms. Front. Oncol..

[25] de Groof A.J., Struyvenberg M.R., van der Putten J., van der Sommen F., Fockens K.N., Curvers W.L., Zinger S., Pouw R.E., Coron E., Baldaque-Silva F. (2020). Deep-Learning System Detects Neoplasia in Patients With Barrett’s Esophagus With Higher Accuracy Than Endoscopists in a Multistep Training and Validation Study With Benchmarking. Gastroenterology.

[26] Souza R., Wang X., Davison J., Goldblum J., Thota P., Duits L., Khoshiwal A., Bergman J., Falk G., Diehl D. (2024). Abstract ID: 81 TissueCypher is the strongest independent predictor of progression in patients with Barrett’s esophagus. Foregut J. Am. Foregut Soc..

[27] Duits L.C., Phoa K.N., Curvers W.L., Kate F.J.W.T., Meijer G.A., Seldenrijk C.A., Offerhaus G.J., Visser M., Meijer S.L., Krishnadath K.K. (2014). Barrett’s oesophagus patients with low-grade dysplasia can be accurately risk-stratified after histological review by an expert pathology panel. Gut.

[28] Sheller M.J., Edwards B., Reina G.A., Martin J., Pati S., Kotrotsou A., Milchenko M., Xu W., Marcus D., Colen R.R. (2020). Federated learning in medicine: Facilitating multi-institutional collaborations without sharing patient data. Sci. Rep..

[29] Ebigbo A., Mendel R., Probst A., Manzeneder J., de Souza L.A., Papa J.P., Palm C., Messmann H. (2018). Computer-aided diagnosis using deep learning in the evaluation of early oesophageal adenocarcinoma. Gut.

[30] World Health Organization (2021). Ethics and Governance of Artificial Intelligence for Health: WHO Guidance.

[31] Tsai M.-C., Yen H.-H., Tsai H.-Y., Huang Y.-K., Luo Y.-S., Kornelius E., Sung W.-W., Lin C.-C., Tseng M.-H., Wang C.-C. (2023). Artificial intelligence system for the detection of Barrett’s esophagus. World J. Gastroenterol..

